# Spatial, temporal, and motivational changes due to the COVID-19 pandemic in a nature-based leisure activity - A global survey of birders

**DOI:** 10.1016/j.isci.2023.107483

**Published:** 2023-07-27

**Authors:** Christoph Randler, Jukka Jokimäki, Maria de Salvo, Renan de Almeida Barbosa, Naomi Staller, Piotr Tryjanowski, Marja-Liisa Kaisanlahti-Jokimäki, Jo-Szu Tsai, Raúl Ortiz-Pulido, Arash Rahafar, Laura Giuffrida

**Affiliations:** 1 Eberhard Karls University Tübingen, Auf der Morgenstelle 24, 72076 Tübingen, Germany; 2 Arctic Centre, University of Lapland, Rovaniemi, Finland; 3 Department of Agriculture, Food and Environment, University of Catania, Catania, Italy; 4 Graduate Program in Science Education, Federal University of Rio Grande do Sul, Porto Alegre, Brazil; 5 Department of Zoology, Poznań University of Life Sciences, Poznan, Poland; 6 Department of Life Science Systems, Technical University of Munich, Freising, Germany; 7 Institute for Advanced Study, Technical University of Munich, Garching, Germany; 8 Department of Biological Resources, National Chiayi University, Chiayi, Taiwan; 9 Population Ecology Laboratory, Autonomous University of the State of Hidalgo, Pachuca, Mexico

**Keywords:** Physical activity, Social sciences

## Abstract

Birdwatchers contribute an immense amount of data to citizen science databases. Thus, birding is important from the leisure perspective and from nature conservation. During the COVID-19 pandemic, we studied birdwatchers on a global scale in over 50 countries by applying the model of behavior change, which focuses on changes in opportunity (spatial, temporal), motivation, and capability (avoidance behavior). The sample consisted of 5051 participants (3437 men, 1575 women, mean age 49 years). Birders changed their spatial behavior to more local birding and to avoidance behavior by choosing different places and different clock times. Concerning motivation, being outdoors showed the highest increase and being with friends the strongest decrease. Higher specialized birders experienced a stronger shift toward regional birding. Birders that focused on new, local, or unrewarding places experienced an increase in motivation. Our study empirically supports the behavior change model and highlights the need to address the heterogeneity of the recreationists.

## Highlights

• We studied 5051 birdwatchers in over 50 countries during the pandemic
• We applied the model of behavioral change on outdoor recreation
• Behavior changed to more local places, more time available, and to avoidance of places
• Birders that focused on new or local places experienced an increase in motivations

## Graphical abstract

**Figure:**
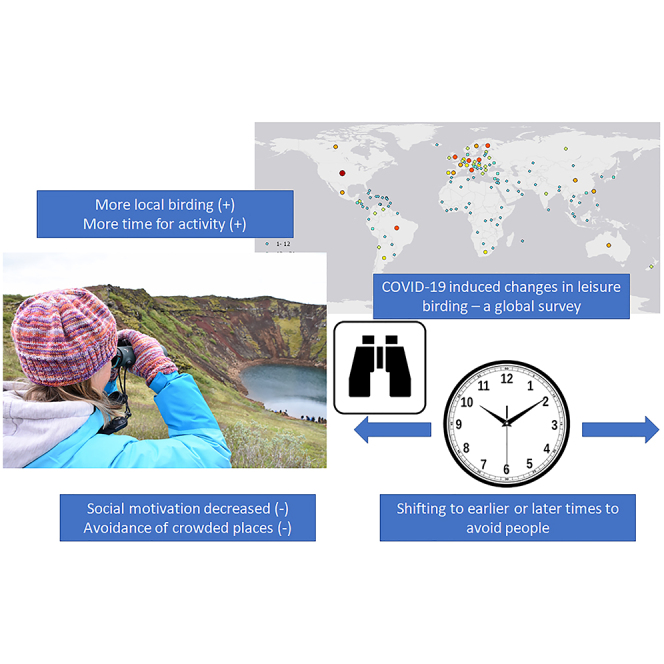

## Introduction

Starting with the first known infection with the novel coronavirus (COVID-19) in December 2019 in Wuhan (China), a global pandemic developed.^1^ In the following period, immense consequences, including governmental activities to restrict the spreading of the virus have taken place. This sudden changes in lifestyle had various physiological (e.g., absence of sports and leisure activities) and psychological consequences (altered sleep, depression, anxiety^2^^,^^3^^,^^4^^,^^5^), as well as a decrease in the mobility of the people.^6^^,^^7^^,^^8^

To address changes in nature-based activities during the pandemic, Soga et al.^9^ (2021) developed a framework considering three lines of research: changes in opportunity, changes in motivation, and changes in capability (based on the study by Michie et al., 2011^10^). Changes in opportunity happen in a temporal and/or spatial context. Temporal changes are related to the possibility to spend more time in a leisure activity due to more available spare time because of remote working at home (and less commuting). Spatial changes are related to changes of mobility because of the governmental lockdown measures, which restrict nature-based activities to the near environment. Changes in motivation are concerned with changes in interest. The third aspect, changes in capability deal with mental and physical resources related to the pandemic and nature experience.

### Changes in spatial and temporal opportunities

Spatial changes in outdoor recreation are often related to more local activities with traveling shorter distances,^11^^,^^12^^,^^13^ and a general shift toward urban birding.^14^ By analyzing eBird data, Hochacka et al.^15^ reported fewer bird observations near wetland habitats and more observations in human-dominated landscapes, suggesting a shift in birding toward less rewarding places in terms of interesting bird species. In Tokyo, Japan, birders visited more often less rewarding birding sites than better sites (with more or rare species) during the pandemic than before.^16^

Temporal changes are related to the time available for leisure activities. US American citizens reported a general increase in home-based traditional leisure and digital/online activities and a decrease in physical and nature-based activities during the pandemic.^17^ In Finland and Sweden, however, greenspace use increased during the pandemic,^13^^,^^18^^,^^19^ while frequency, duration, and quality of nature interactions decreased in Israel.^20^ Accordingly, in population-based studies in Vermont, USA, wildlife watching as a leisure activity increased,^21^^,^^22^ and in Flanders, Belgium, nature visits increased in general.^23^ Concerning citizen science data collection of biodiversity, a decrease of 60% between 2019 and 2020 was found in Tokyo, Japan.^16^ However, Basile et al.^24^ found an increase in daily bird observations in urban areas in Spain and Italy, and a decrease in rural areas in Spain and Italy. Another aspect might be a temporal change described by Randler et al.,^2^ where birders reported to go birdwatching at different time slots compared to the pre-pandemic situation (i.e., either earlier or later), partly because of more available time and working at home, but also to avoid contact with other people. Thus, in sum, changes in opportunities may have led to a higher amount of time available for birding (temporal aspect) and to birding visits in more local, less rewarding places, while skipping international travel (spatial aspect).

### Changes in motivation

Changes in motivation have been described only in a few studies. For the general public in Flanders, Belgium, Lenaerts et al.^23^ reported a higher interest into nature during the pandemic. Similar results have been obtained from Finland.^13^ Concerning nature-based leisure activities in the USA, about 30% of anglers reported a change in their motivation for fishing during the pandemic, and stress relief was a more popular motivation during the pandemic than before.^25^ In the USA, data submitted to eBird increased on a nationwide scale, indicating increased motivation to go for birding.^14^ In a citizen science project in Tokyo,^16^ enthusiastic naturalists contributed to similar amounts of time before and during the pandemic, but the participation by less enthusiastic volunteers declined drastically during the pandemic. Motivations in birding have been studied in many aspects in the pre-pandemic (see overview in^26^), but to our knowledge, no study has yet addressed this question in relation to the pandemic.

### Changes in capabilities

Social and psychological aspects are related to the third pathway, i.e., to changes in capability.^9^ In Finland, preliminary results suggest that nature-liked areas in cities and elsewhere have been worked as important sites for increasing well-being during the pandemic.^13^ In the UK, interactions with non-companion animals (e.g., wildlife) and frequent contact with nature had a positive impact on mental health during the pandemic.^27^ In Israel, environmental attitudes and affinity toward nature remained similar before and during the lockdown.^20^ Concerning US American birders, Randler et al.^28^ analyzed their sentiments in an open question and found that these became more negative in the second wave of the pandemic. Therefore, the progress of the pandemic and its duration significantly negatively influenced well-being. In addition, social outdoor recreational activities decreased during the pandemic,^22^ and respondents generally considered such activities as rather safe, when they were carried out as singletons.^25^

### Demographic aspects

The pandemic had a differential impact on the female and male population, e.g., in areas of occupational success (e.g., women achieved fewer academic milestones than men during the pandemic),^29^ employment (more women than men lost their jobs during the pandemic^30^), the double burden of work and childcare,^31^^,^^32^ and health (e.g., the prevalence of domestic violence against women increased significantly due to the lockdown measures).^33^ In general, women had a greater decline in well-being than men during the pandemic.^34^ There seems to be also clear gender differences in leisure activities. Working mothers were significantly restricted in their leisure time and physical activity due to the double burden of work and childcare, while fathers showed less change.^35^ Women were also more cautious in their social interactions. For example, women who spent their free time birdwatching reported canceling trips significantly more often than men with the same hobby.^2^

The age of the population had a significant impact on people’s life and behavior during the COVID-19 pandemic. Younger people were most affected by the economic situation and effects on their psyche.^36^ In contrast, older people assessed the risk associated with COVID-19 significantly higher than younger people.^37^ For example, in Slovakia, people aged 16–29 visited the forest about 20% more than before the lockdown, whereas in the age groups 30–44 and 45–62, forest visits also increased significantly.^38^ Similarly, Venter et al.^19^ showed a 4-fold increase in outdoor activity data for teenagers in Norway. Also, in Finland, especially young people (14–24 years old) reported that they have found a new outdoor hobby during the pandemic.^13^

### Birding specialization

Previous COVID-19 studies have addressed mainly the health effects, usually in a population-based manner (i.e., addressing the general public), but only rarely the studies have analyzed a specialized leisure activity, such as birding or recreational fishing.^2^^,^^25^^,^^28^ Further, to our best knowledge, no studies have considered the possible influence of individual variance on human-nature interactions during the pandemic. Individual variance might influence the observed results related to changes in opportunity, changes in motivation, and changes in capability. As there are many different types of birders,^39^ it is especially important to take this variance into account when analyzing the effects of COVID-19 on behavior of birders. Birding specialization addresses the individual variation among birders and is based on three dimensions.^40^ First, skill/knowledge covers the ability to identify birds by sight and sound without any aid (book, app). Second, behavior portrays the birding behavior, e.g., the number of field trips and outings. Third, commitment or centrality to lifestyle addressed a psychological construct, e.g., the importance of this leisure activity for ones’ own life.

### The current study

In our study, we focused on the concept of birding specialization and motivations. Motivations for birdwatching follow different psychological concepts,^26^ such as enjoying the sights/sounds of nature, to be outdoors, or to see as many bird species as possible.^41^ These aspects have never been addressed with respect to a nature-based outdoor recreational activity during the COVID-19 pandemic. The main aim of this study is to globally analyze changes in birdwatching or birding behavior due to the pandemic. Birding is a nature-based outdoor leisure activity that is related to the spotting and observing of birds and carried out by millions of people around the globe.^42^ Further, birders contribute an immense amount of data to publicly available datasets that help to monitor bird numbers and contribute to bird conservation.^43^ Thus, birding is interesting from the leisure perspective, but also from the nature conservation viewpoint. In this study, we applied the three-dimensional model proposed by Soga et al.^9^ to our global birding data by using an explorative factor analysis. We analyzed the relationship between birding specialization and the pandemic on the one side, and the changes in opportunity, motivations, and capability^9^ on the other side on a global scale and considering possible demographic factors simultaneously.

We hypothesize that the specialization level of birders will influence how birders will change their birding activities. We predict that highly specialized birders, i.e., birders with a high level of commitment, are more likely to feel restricted in their birding activities by different kinds of restrictions than less involved birders. Further, we hypothesize that restrictions would impact differently on men and women as well as on different age groups. We predict that men and younger birders will be less affected by COVID-19 restrictions than women and older birders since men and younger people are more risk-taking than women and older people.^2^ The study is unique since we did not use secondary data (like the analysis of eBird entries), but rather asked the people directly about their behavioral change.

## Results

**Figure 1:**
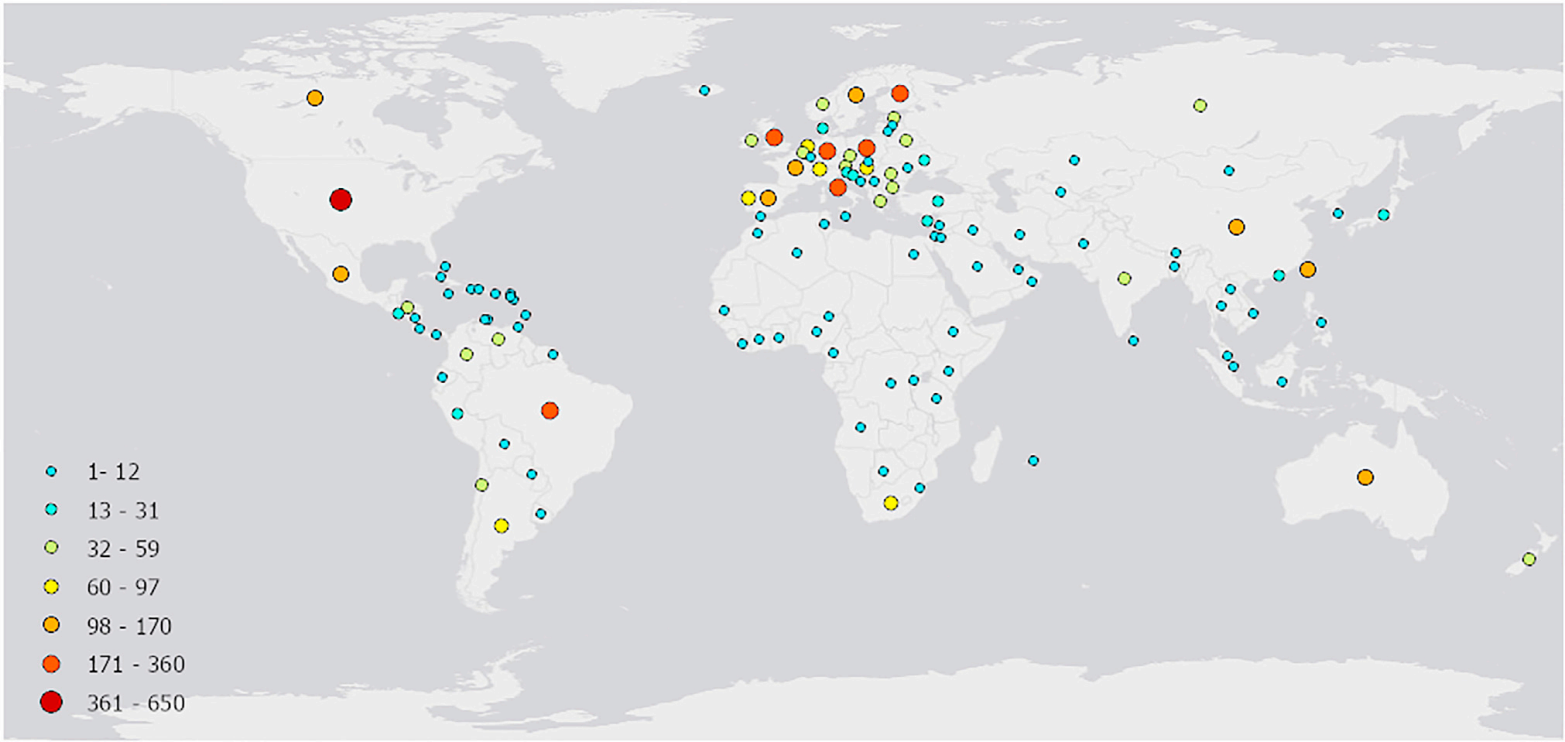
Global distribution and numbers of respondents per different countries (n = 5051)

A total of 5051 participants responded to our questionnaire (Figure 1). The sample comprised 3437 men (68%), 1575 women (31%), 14 non-binary, and 25 who preferred not to answer. Mean age was 48.99 years ±16.22. Women and men did not differ in age from another (t test: t = −0.260, df = 4972, p = 0.795). About 21.6% worked in a remote working situation, 20.9% at their usual workplace, 19.0% in a hybrid model, while 6.9% reported to be unemployed and 5% were students. 19.7% reported to be pensioners, and 7% did not report their current working situation.

### General view for the spatial changes in birding

Most participants reported spatial changes in their birding behavior, with 61% (n = 3070) going more often to local places (shorter traveling distances) than previously. Similarly, 39% reported to do more home-based birding, like observing birds at feeders, making garden lists of observed birds or watching birds from the window during pandemic compared to earlier years. Only 5% indicated that they watch birds less in their homes during the pandemic than earlier. Also, about 72% responders indicated that they went to less rewarding places at least sometimes during the pandemic.

### General view for the temporal changes in birding

Nearly, a third reported that they have increased, and another third indicated that they have decreased birding during the pandemic. Related to the time spend in bird-related topics on the internet, 35% birders (n = 1747) reported to have spent more time on the internet during the pandemic than previously. Concerning data collection for monitoring programs, a total of 1350 responders indicated that they did not participate in monitoring programs, 1025 birder reported that the pandemic strongly influenced their data collection in a negative manner, 1221 in a little manner, and 1257 stated that the pandemic did not influence in any way their data collection.

### General view for the motivational changes in birding

Changes in motivation were rather weak; most birders reported no changes in their birding motivation (Table 1), but “being outdoors” showed the highest increase during the pandemic. However, the item “… to be with friends” was reported to become a less important factor going for birding during the pandemic as reported by about 40% (Table 1).

**Table 1:** Changes in birding motivations during the COVID-19 pandemic

		enjoy being alone		be outdoors		enjoy the sights/sounds of nature		to be with friends		get away from the demands of life		… to see as many bird species as possible
	N	%	N	%	N	%	N	%	N	%	N	%
Less during COVID-19 than before	423	8.40%	484	9.60%	370	7.30%	1979	39.2	407	8.10%	840	16.60%
No changes	3511	69.50%	2721	53.90%	3092	61.20%	2466	48.8	3206	63.50%	3219	63.70%
More during COVID-19 than before	891	17.60%	1618	32.00%	1373	27.20%	360	7.1	1199	23.70%	770	15.20%
Missing cases	226	4.50%	228	4.50%	216	4.30%	246	4.9	239	4.70%	222	4.40%

The items are based on the study by Hvenegaard (2002) and asked for a perceived change during the pandemic. Less during COVID-19 means that this motivational aspect decreased during the pandemic; more during COVID-19 means that this motivational dimension increased during the pandemic.

### General view for the capability changes in birding

In line with avoidance behavior, 52% of the respondents reported changes in their birding by avoiding overcrowded places such as birding towers (n = 2620). About 20% reported some circadian changes in their birding activity; they go birding either earlier or later than before the pandemic to avoid meeting others (n = 1009).

### Results of the PCAs

#### PCA-based changes in birding opportunities and capability

The principal component analysis (PCA) with varimax rotation revealed three factors with an eigenvalue greater than one: PC1 1.654 (23.6% variance explained), PC2 1.179 (16.8%), and PC3 1.030 (14.7%; Table 2). PC1 is considered with less rewarding birding, such as home birding at the feeder, visiting places that are less rewarding, and places that are more local. Thus, it is labeled “*spatial changes in birding*”. Higher scores represent a shift toward more restricted and local birding. PC2 is labeled as “*temporal changes in birding*” and it combines the items dealing with a higher amount of birding in general, in line with a higher amount of internet birding. Thus, a higher score represents a higher amount of time spent in birding activity—birding on the internet thus does not go toward the costs of spending less time outside. PC3 describes the avoidance of overcrowded birding places and also temporal changes which may be related to a change in activity to avoid contact with others (Table 2). Thus, the PC3 was labeled as “*avoidance behavior*”.

**Table 2:** Factor loadings of the principal component analysis with varimax rotation

	PC1 Spatial changes in birding	PC2 Temporal changes in birding	PC3 Avoidance behavior
I go to local places more. [coded: 0 = no, 1 = yes]	**0.743**	0.122	−0.058
I currently go to less rewarding or under-birded places (in terms of species or scenery). [-2 = never, −1 = seldom, 0 = same, 1 = often, 2 = always]	**0.719**	−0.179	0.173
I currently do home birding (feeder/yard/from window) [-1 = less, 0 = same, 1 = more]	**0.511**	0.266	0.099
My time spent birding has … [-1 = decreased, 0 = same, 1 = increased]	0.083	**0.770**	−0.127
Regarding the time spent surfing “bird-related” topics in the internet (e.g., facebook, eBird, ornitho.de etc.) [-1 = less time, 0 = same, 1 = more time]	0.048	**0.709**	0.195
I avoid popular birding places, like bird towers, due to COVID-19. [0 = no, 1 = yes]	0.130	−0.111	**0.768**
I changed the clock time I go birding to either earlier in the morning or later in the evening compared to before the pandemic to avoid other people. [0 = no, 1 = yes]	0.023	0.185	**0.748**

Loadings in bold show the relation to the respective factor.

There was a significant correlation between the spatial change (PC1) and birding specialization (r = 0.131, p < 0.001, N = 5051), thus higher specialized birders experienced a stronger shift toward regional and local birding.

**Figure 2:**
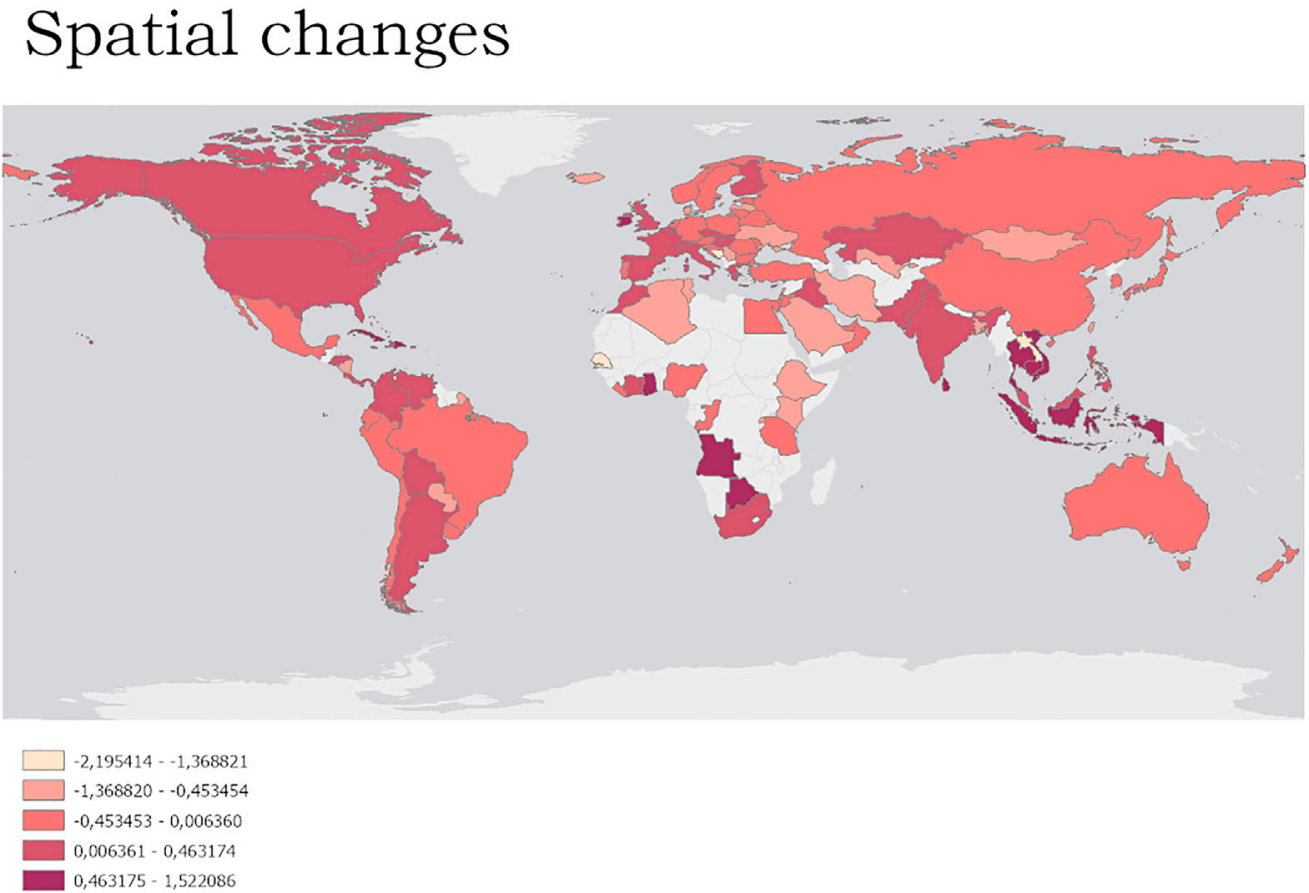
Spatial changes in birding based on the PC1 scores Higher scores (darker red color) represent a more local birding (at home; more local; and in less rewarding places).

**Figure 3:**
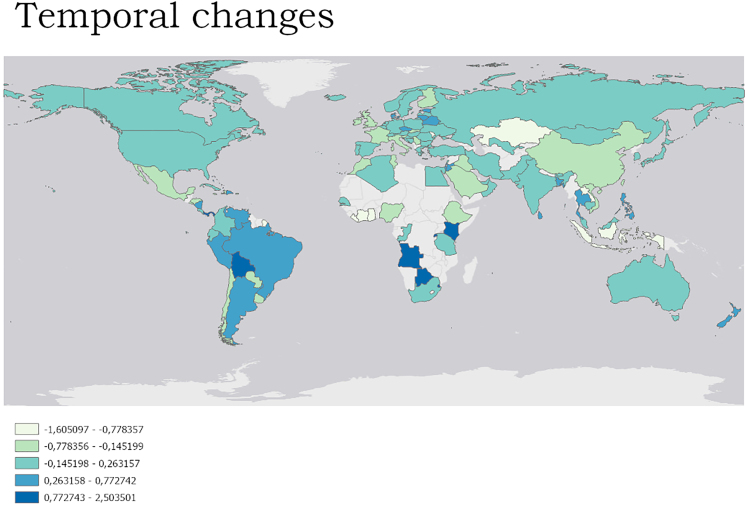
Temporal changes in birding based on the PC2 scores Higher scores (blue color) = more time for birding.

Spatial changes (more local/home birding) in birding were reported especially in some African and Asian countries as well as in the USA, whereas less spatial changes were observed in European countries (Figure 2). Temporal changes (PC2) in birding, with less time spend for the birding, were reported especially in European countries as well as USA, whereas more time for birding was reported in some South American and African countries (Figure 3).

**Figure 4:**
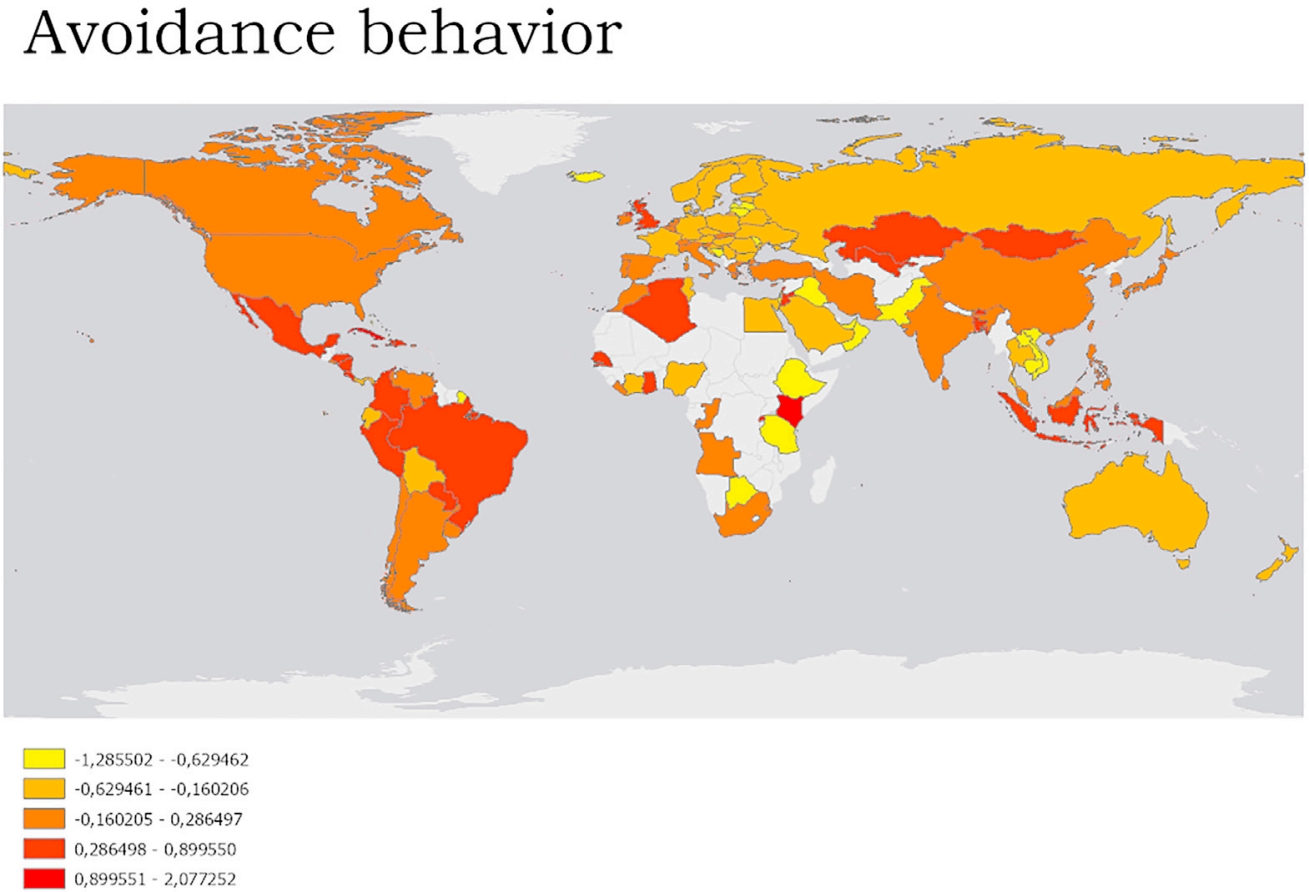
Avoidance behavior based on the PC3 scores Higher scores (red color) = more avoidance behavior.

Avoidance behavior (PC3) of overcrowded birding sites was reported in e.g., parts of South America, whereas birders in Europe and Australia reported less avoidance behavior (Figure 4).

#### PCA-based changes in birding motivation

The first principal component analysis with varimax rotation related to changes in birding motivation revealed two factors with eigenvalues 2.612 and 1.041. However, there were severe cross-loadings, and the second component consisted only of the item “… to be with friends.” Hence, this item was dropped from the scale for further analyses (descriptive aspects concerning this item are shown in Table 2). Subsequently, the remaining five items loaded onto the same single factor (PC1), with an eigenvalue of 2.542 and 50.8% of variance was explained (Table 3). Higher scores on the birding motivation scale showed that birding motivation has increased during the pandemic. Therefore, the PC1 was labeled as “*motivational change*”.

**Table 3:** Factor loadings of the change in birding motivation

I go birding to …	PC1 Motivational change
… enjoy the sights/sounds of nature	0.820
…. be outdoors	0.802
… get away from the demands of life	0.692
… see as many bird species as possible	0.649
… enjoy being alone	0.571

Motivational change was related to shift in behavior. The spatial change toward unrewarding and more local places was positively correlated with birding motivation, i.e., the birders that focused their birding on new, local, or unrewarding places also experienced an increase in motivation (Table 4). Similarly, the shift toward more time spent birding was positively correlated with motivation.

Motivational change was related to shift in behavior. The spatial change toward unrewarding and more local places was positively correlated with birding motivation, i.e., the birders that focused their birding on new, local, or unrewarding places also experienced an increase in motivation (Table 4). Similarly, the shift toward more time spent birding was positively correlated with motivation.

**Table 4:** Relationship between changes in opportunity and motivational change and data submission to web-based platforms

		Motivational change (n = 5051)	Submission of data (n = 5050)
Spatial change in birding	Pearson’s r	0.110	0.144
	P	<0.001	<0.001
Temporal change in birding	Pearson’s r	0.342	0.076
	P	<0.001	<0.001
Avoidance behavior	Pearson’s r	−0.034	−0.058
	P	0.014	<0.001
Birding specialization	Pearson’s r	−0.02	0.463
	P	0.149	<0.001

Concerning data collection for citizen science projects, data collectors contributing a high amount of data experienced a spatial change in birding more than collectors with fewer data submissions. Thus, birders more active in citizen science projects and on web-based platforms only slightly changed their temporal behavior (with more time available) but more strongly changed their spatial behavior with more time birding in closer localities (Table 4). Birding specialization itself was medium-sized correlated with submitting data.

### Results of the GLM analyses

According to the general linear mixed model analyses, birding specialization was related to a spatial and temporal change, but not to motivational change and avoidance behavior (Table 5). The effect size related to temporal change was negligible. Age effects were significant, but with a small effect size <0.01, except the relationship between age and motivational change (Table 5). Thus, older people experienced a decrease in motivation during the pandemic. Finally, gender effects were found in temporal change, avoidance behavior, and motivational change (Table 5). Women showed a higher value concerning their temporal change during COVID-19 (estimated mean ± se: men −0.040 ± 0.027; women: 0.144 ± 0.042). Thus, birding opportunities have increased for women. Concerning avoidance behavior, women scored higher than men (men: −0.054 ± 0.027; women: 0.061 ± 0.041), suggesting that women exhibited a higher avoidance behavior. Birding motivation decreased significantly stronger in men than in women (men: −0.108 ± 0.027; women: 0.019 ± 0.041). A significant and sizable effect of country was found in all four models (Table 5).

**Table 5:** Results of the general linear mixed models; the four outcome variables (spatial change, temporal change, avoidance behavior, and motivational change) as dependent variables, gender as fixed factor, age and birding specialization as covariates, and country as random factor

	Spatial changes in birding				Temporal changes in birding				Avoidance behavior				Motivational change in birding			
Source of variance	MS	F	P	Partial Eta-squared	MS	F	P	Partial Eta-squared	MS	F	P	Partial Eta-squared	MS	F	P	Partial Eta-squared
Constant	23.766	22.798	<0.001	0.012	45.509	43.777	<0.001	0.024	6.035	5.504	0.019	0.006	46.125	48.158	<0.001	0.017
Age	13.579	14.695	**<0.001**	0.003	59.387	64.573	**<0.001**	0.014	15.620	17.597	**<0.001**	0.004	132.302	148.28	**<0.001**	0.031
Birding specialization	68.099	73.699	**<0.001**	0.016	4.870	5.295	**0.021**	0.001	0.148	0.167	0.683	0.001	0.965	1.081	0.299	0.000
Gender	0.665	0.782	0.377	0.002	11.653	10.442	**0.001**	0.053	6.576	6.509	**0.011**	0.030	5.571	5.674	**0.018**	0.024
Country	4.488	5.962	**<0.001**	0.848	4.517	3.276	**<0.001**	0.763	7.161	6.099	**<0.001**	0.856	2.859	2.595	**<0.001**	0.716
Gender ∗ country interaction	0.745	0.807	0.833	0.009	1.399	1.521	**0.011**	0.016	1.187	1.337	0.057	0.014	1.111	1.245	0.116	0.013

Note: MS = Mean of squares.

Significant main effects are indicated in bold.

## Discussion

In general, our study gave empirical support for the model of outdoor recreation during the pandemic developed by Soga et al.^9^ (based on Michie et al.^10^) Our results clearly indicated the three pathways of human-nature interaction, i.e., changes in opportunity, changes in motivation, and changes in capability identified by Soga et al.^9^ First, our results indicated changes in opportunity since birders changed their spatial and temporal behavior. Second, birders reported changes in motivation since with a decrease in social motives. And lastly, our data indicated that the pandemic caused changes in capability to practice birding hobby, e.g., by exhibiting avoidance behavior in terms of avoiding other people by choosing different places and different clock times for birding. In addition, our results demonstrated that behavioral changes among birders due to the COVID-19 pandemic were depending on specialization level of their hobby as well as demographic factors.

### Spatial, temporal, and motivational changes in birding

Our data indicated that birders observed birds more often closer to their homes or even stayed at home to watch birds in their gardens or from the windows. Alternatively, some birders were practicing their hobby on the internet during the pandemic more intensely than previously. Such a trend to nearer places has been revealed by visitors of urban and semi-urban natural areas in Vermont^21^ and in Finland.^13^ Rice at al.^44^ showed a decrease of distance traveled to participate in outdoor recreation from 5 to 3 km. The possibility to visit nearby local natural areas is an important aspect for health-related quality of life. For citizen science data collection projects, this means that new places may be discovered, and that data may be more evenly distributed compared to the focus of birders on highly rewarding hotspots with many species. By avoiding overcrowded sites, the discovery of new and valuable or rarely visited sites may increase data quality.

Our results indicated that birders that focused their birding on new, local, or unrewarding places also experienced an increase in their motivation. This can be explained because they may have explored new terrain and tried to make the best out of this situation by following the governmental measures and restrictions without pausing in birding. The discovery of a new birding site may be an exciting experience and could be more rewarding than just visiting the usual, overcrowded places. The birds detected at a less watched place may be worth “more” when discovered by oneself rather than “following the crowd” when searching for species discovered by others (e.g., in the case of twitching).

### Capability changes in birding

We found changes in avoidance behavior, especially the avoidance of overcrowded places like bird towers, but also the avoidance of usual birding times. This means that people shifted their behavior to clock times that may be less rewarding, e.g., when shifting their birding activity from morning to midday. However, even the opposite may happen, namely shifting to an earlier time (e.g., during the sun rise) which could even improve birding opportunities since birds are then most active and are therefore easier to detect, e.g., by their song. However, this remains speculative since we have no direct data on this. Further, our results indicated that social birding has significantly decreased during the pandemic. This was inferred indirectly from the changes in motivations, because the motivation “to be with friends” has significantly decreased during the pandemic and has lost importance, similar to other non-birding studies.^21^^,^^44^

Stenhouse et al.^45^ showed that stricter and longer restrictions reduced the numbers of scientific observations. Our results, in concordance with others, suggest that the citizen science community remained active during the lockdowns and kept reporting birds from home or from places near their homes. Specialized birders reported a stronger shift toward local birding than less specialized birders. This can be explained by the commitment and the centrality to lifestyle component of the birding specialization construct.^40^ Birders that are highly committed to birding cannot stop their behavior, and if travel restrictions are applied by the government, highly specialized birders shift toward the local environment or even to home/garden birding. Birding motivations is usually positively related to birding specialization,^26^ but we found no evidence for this in our data. This might be due to the fact that we did not assess birding motivations itself, but rather changes in motivations due to the pandemic.

### Influences of birding specialization

Birding specialization was found to have a strong impact on our results. That is, the skill/knowledge, behavior, and the psychological commitment played an immense role. Higher specialized birders submitted more data to web-based platforms and citizen science projects in general^43^ and during the pandemic (current study). Also, higher specialized birders reported a stronger spatial shift toward more local and less rewarding places. This suggests that keen birders did not change their birding activity but tried to keep on birding by fulfilling the governmental restrictions. Interestingly, despite the strong restrictions, motivational change was unrelated to birding specialization. One might expect that restrictions may affect specialized birders stronger because there were restrictions on travel and on the circumference for leisure activities.

### Demographic factors

The age of the respondents influenced how the pandemic changed birders’ spatial and temporal birding behavior, avoidance of overcrowded places, and motivations to go birding. Older birders tended to a higher avoidance behavior and to stronger restrictions concerning their spatial and temporal habits. These effects can be explained from a personality point of view since older people are risk-aversive and have a higher anxiety. We here replicate the findings from a previous qualitative study.^2^ This may also explain why the motivation decreased stronger in older respondents.

We found gender-specific differences in how the pandemic influences the temporal changes, avoidance, and motivation aspects of birding. The opportunity for women to spend more time in birding seems to have increased more than in men. Similarly, in Finland, more woman than men have reported that they appreciated more their closest nature during the pandemic than the pre-pandemic period.^13^ Thus, the opportunities to experience nearby nature may have been higher in women. Also, motivation decreased stronger in men than in women, even when controlling for birding specialization. This is difficult to explain, and there are no supporting data on this. Women showed a stronger avoidance behavior, which is in line with our previous qualitative study.^2^

### Limitations of the study

In addition to birding, we might have asked many additional questions on the individual level, such as personality correlates or about resilience. However, we tried to keep the numbers of questions reasonable to achieve a high compliance. Our sample is partly biased on European and OECD countries, whereas we had few participants from the Global South. However, our data contained quite large material also from Brazil and South Africa. Further, our sampling methods may have caused a bias toward higher specialized birders and is not representative for the general public. Nevertheless, the intention was to sample birders. Hochacka et al.^15^ studied real behavior of eBird participants during the pandemic by analyzing their data entries. Their study is partly complementary to our study, in several ways. First, because it analyses real behavior by a data analysis compared to our survey, and, second, it is focused only on eBird participants, while our study covers a quite wide range of birdwatchers. Many birdwatchers are not submitting data to citizen science platforms.^43^ For example, in German birdwatchers, only 38% were participating in the German ornitho.de citizen science data collection and the others not. In this current study, about 20% of the respondents never reported sightings to such citizen science platforms. Therefore, our study also focuses on a more diverse sample of birdwatchers than previous work. Our study gives some venues for the further research. For example, it would be interesting to compare our birder-related results for the results of other outdoor recreation groups, e.g., recreational fishing or nature photography. Also, it would be valuable to study how quickly birding behaviors will recover to the normal state.

## STAR★Methods

### Key resources table

**Table:** 

REAGENT or RESOURCE	SOURCE	IDENTIFIER
**Deposited data**		

Raw and analyzed data	This paper	osf.io/ucyqk

**Software and algorithms**		

SPSS 28	IBM SPSS statistics	https://www.ibm.com/de-de/products/spss-statistics

### Resource availability

#### Lead contact

Further information and requests for resources and reagents should be directed to and will be fulfilled by the lead contact, Christoph Randler (Christoph.randler@uni-tuebingen.de).

#### Materials availability

This study did not generate new unique reagents.

### Experimental model and study participant details

#### For studies involving human participants

##### Sampling procedure

We aimed to sample a large and broad population of birders around the globe. The main strategy of the search was to contact national groups and organizations and websites that promote birding and bird conservation. Then, the search was widened to regional groups and organizations, such as federal states, county, and regional and local birding societies or groups. We considered both, formal organizations as well as informal bird clubs. Websites of bird magazines or bird clubs were used, and overview websites, such as fatbirder.com and birdguides.com and others provided plenty of contact information. The search strategy was identical across the regions, but regions differ in their birdwatching activities and in their structure of ornithological societies and birding clubs. Then, participants were recruited via many channels, e.g., using announcements placed on the webpages of large bird and nature-based organizations. Mailing lists were used from some organizations. Scientific ornithological unions, societies and clubs were asked for participation by using postings on their websites or by distribution the link on their newsletter or mailing lists. In addition, more than 100 international Facebook groups with a relation to birdwatching were used to post an information about the study. Data were collected with the Online Research Tool SoSciSurvey. Data collection was carried out between December 19^th^ 2020 and April 16^th^ 2021. The questionnaire was available in 10 languages (English, German, Polish, Finnish, Italian, Portuguese, Spanish, Russian, Greek and Chinese (traditional and simplified). On the first page of the survey, the aims of the study were explained, and a formal informed consent was requested. Participants had to actively click on “yes” to start the study. They were also able to stop and leave at any times without any consequences. The study was granted permission by the ethics committee of the Poznan Life Science University [27.4.2020, waiver, following the Act on the Protection of Animals used for Scientific or Educational Purpose in Poland].

The influence (or association) of sex, gender, or both on the results of the study are reported in the results section. Information related to the participants (e.g., sample size, etc.) is reported in the methods and results section. The committee approving the studies is mentioned in the methods section.

### Method details

#### Questionnaires

##### Changes in opportunity

Following Soga et al.^9^ we used items to measure change in birding opportunities that have been developed explicitly for this current survey based on the results of a previous qualitative study.^2^ These items contain questions about spatial and temporal changes, avoidance behavior and others.

##### Changes in motivation

To measure changes in birding motivations, we adapted the scale from Hvenegaard^41^ dealing with motivations for birding. The six items for the motivation questions were based on previous studies (see Table 1). The scale was constructed to capture the change in birding motivation related to COVID-19. The scales were coded from −1 = less during COVID-19, 0 = remaining the same, and 1 = more during COVID-19. Higher scores represented higher motivational change. None of the items was reverse coded. Cronbach’s α was 0.755 after dropping the item “… be with friends” (see below).

##### Changes in capability

Changes in capability were related to social and psychological aspects, especially in avoidance behavior. The items dealt with avoidance of time slots and with avoidance of crowding.

##### Birding specialization

Birding specialization was based on a measurement developed by Lee and Scott,^46^ subsequently translated into different languages by the authors of this study. The scale consists of 9 items: three ask for the skill/knowledge (how many bird species one can identify by sight and sound, self-assessment of birding skills), two ask for behavior (field trips and field days), four ask about centrality to lifestyle and psychological commitment. Birding specialization was operationalized to the pre-pandemic situation. Cronbach’s α for the total sample was 0.791.

##### Demographics

Demographics (age in years and gender: male, female, diverse, prefer not to answer) and country of actual residence were collected. Further, we asked whether people regularly submit bird observations to web-based citizen science platforms, such as eBird, observando or ornitho. Answers provided were: almost daily, several times per week, weekly, monthly, less than monthly, and never (higher scores were related to fewer submissions and were recoded for the results table). All datasets and methods are available at https://doi.org/10.17605/OSF.IO/UCYQK.

### Quantification and statistical analysis

All statistical analyses were carried out with SPSS 28 (see key resources table). Please note that not all respondents answered all questions. Therefore, we always give the exact degrees of freedom or sample size. For comparison of two samples (gender differences), we used a Student’s *t* test. As we have developed items for measuring change in birding activity based on the previous qualitative survey^2^ (Randler et al., 2020), we applied an exploratory factor analysis (principal component) with a varimax rotation to estimate, which items fit together into a scale. The factor scores have been saved as residuals (z-scores) for further analyses. We used the derived PCA factors to study the relationships between the changes in opportunities, motivations and capability in birding, and demographic effects. Pearson`s correlation (r) was used to measure the relationships between variables. For the general linear mixed model, we used only countries with more than 10 respondents. We ran a series of four general linear mixed models (GLM) for the four outcome variables describing changes in opportunities (1. spatial change and 2. temporal change), 3. changes in motivations and changes in capability (4. avoidance behavior). Gender was used as fixed factor, age and birding specialization as covariates and country as random factor in all four GLMs. In addition, the interaction term gender x country was included to assess whether gender patterns are similar across countries. Data are available in the Open Science Framework: osf.io/ucyqk. Significance was defined as p < 0.01. All statistical calculations are available in the open science framework: https://doi.org/10.17605/OSF.IO/UCYQK.

## Data Availability

The raw questionnaire data for both analysis (scale development; country analysis) have been deposited at Open Science Framework and are publicly available as of the date of publication. Accession numbers are listed in the key resources table. The DOI is https://doi.org/10.17605/OSF.IO/UCYQK. All original code has been deposited at Open Science Framework and is publicly available as of the date of publication. https://doi.org/10.17605/OSF.IO/UCYQK. Any additional information required to reanalyze the data reported in this paper is available from the lead contact upon request.

## References

[1] Wu Z., McGoogan J.M. (2020). Characteristics of and important lessons from the coronavirus disease 2019 (CoVID-19) outbreak in China: summary of a report of 72 314 cases from the Chinese center for disease control and prevention. JAMA.

[2] Randler C., Tryjanowski P., Jokimäki J., Kaisanlahti-Jokimäki M.L., Staller N. (2020). SARS-CoV2 (COVID-19) Pandemic lockdown influences nature-based recreational activity: The case of birders. Int. J. Environ. Res. Publ. Health.

[3] Lai J., Ma S., Wang Y., Cai Z., Hu J., Wei N., Wu J., Du H., Chen T., Li R. (2020). Factors associated with mental health outcomes among health care workers exposed to coronavirus disease 2019. JAMA Netw. Open.

[4] Staller N., Randler C. (2022). Chronotype and Organizational Citizenship Behavior during the CoVID-19 restriction phase in Germany. Biol. Rhythm. Res..

[5] Zhang C., Yang L., Liu S., Ma S., Wang Y., Cai Z., Du H., Li R., Kang L., Su M. (2020). Survey of insomnia and related social psychological factors among medical staffs involved with the 2019 novel coronavirus disease outbreak. Front. Psychiatr..

[6] Reisch T., Heiler G., Hurt J., Klimek P., Hanbury A., Thurner S. (2021). Behavioral gender differences are reinforced during the COVID-19 crisis. Sci. Rep..

[7] Ugolini F., Massetti L., Calaza-Martínez P., Cariñanos P., Dobbs C., Ostoić S.K., Marin A.M., Pearlmutter D., Saaroni H., Šaulienė I., Sanesi G. (2020). Effects of the COVID-19 pandemic on the use and perceptions of urban green space: An international exploratory study. Urban For. Urban Green..

[8] van Leeuwen M., Klerks Y., Bargeman B., Heslinga J., Bastiaansen M. (2020). Leisure will not be locked down –insights on leisure and COVID-19 from the Netherlands. World Leis. J..

[9] Soga M., Evans M.J., Cox D.T.C., Gaston K.J. (2021). Impacts of the COVID-19 pandemic on human–nature interactions: Pathways, evidence and implications. People Nat..

[10] Michie S., Van Stralen M.M., West R. (2011). The behaviour change wheel: a new method for characterising and designing behaviour change interventions. Implement. Sci..

[11] Korpilo S., Kajosaari A., Rinne T., Hasanzadeh K., Raymond C.M., Kyttä M. (2021). Coping With Crisis: Green Space Use in Helsinki Before and During the COVID-19 Pandemic. Front. Sust. Cities.

[12] Mul E., Ancin Murguzur F.J., Hausner V.H. (2022). Impact of the COVID-19 pandemic on human-nature relations in a remote nature-based tourism destination. PLoS One.

[13] Neuvonen M., Lankia T., Kangas K., Koivula J., Nieminen M., Sepponen A.-M., Store R., Tyrväinen L. (2022).

[14] Crimmins T.M., Posthumus E., Schaffer S., Prudic K.L. (2021). COVID-19 impacts on participation in large scale biodiversity-themed community science projects in the United States. Biol. Conserv..

[15] Hochachka W.M., Alonso H., Gutiérrez-Expósito C., Miller E., Johnston A. (2021). Regional variation in the impacts of the COVID-19 pandemic on the quantity and quality of data collected by the project eBird. Biol. Conserv..

[16] Kishimoto K., Kobori H. (2021). COVID-19 pandemic drives changes in participation in citizen science project “City Nature Challenge” in Tokyo. Biol. Conserv..

[17] Shen X., MacDonald M., Logan S.W., Parkinson C., Gorrell L., Hatfield B.E. (2022). Leisure engagement during COVID-19 and its association with mental health and wellbeing in US adults. Int. J. Environ. Res. Publ. Health.

[18] Beery T., Olsson M.R., Vitestam M. (2021). COVID-19 and outdoor recreation management: Increased participation, connection to nature, and a look to climate adaptation. J. Outdoor Recreat. Tour..

[19] Venter Z.S., Barton D.N., Gundersen V., Figari H., Nowell M.S. (2021). Back to nature: Norwegians sustain increased recreational use of urban green space months after the COVID-19 outbreak. Landsc. Urban Plann..

[20] Colléony A., Clayton S., Shwartz A. (2022). Impacts of nature deprivations during the COVID-19 pandemic: A pre-post comparison. Biol. Conserv..

[21] Grima N., Corcoran W., Hill-James C., Langton B., Sommer H., Fisher B. (2020). The importance of urban natural areas and urban ecosystem services during the COVID-19 pandemic. PLoS One.

[22] Morse J.W., Gladkikh T.M., Hackenburg D.M., Gould R.K. (2020). COVID-19 and human-nature relationships: Vermonters’ activities in nature and associated nonmaterial values during the pandemic. PLoS One.

[23] Lenaerts A., Heyman S., De Decker A., Lauwers L., Sterckx A., Remmen R., Bastiaens H., Keune H. (2021). Vitamin Nature: How Coronavirus Disease 2019 Has Highlighted Factors Contributing to the Frequency of Nature Visits in Flanders, Belgium. Front. Public Health.

[24] Basile M., Russo L.F., Russo V.G., Senese A., Bernardo N. (2021). Birds seen and not seen during the COVID-19 pandemic: The impact of lockdown measures on citizen science bird observations. Biol. Conserv..

[25] Midway S.R., Lynch A.J., Peoples B.K., Dance M., Caffey R. (2021). COVID-19 influences on US recreational angler behavior. PLoS One.

[26] Randler C., Großmann N. (2022). Motivations for birdwatching scale–Developing and testing an integrated measure on birding motivations. Front Bird Sci.

[27] Shoesmith E., Shahab L., Kale D., Mills D.S., Reeve C., Toner P., Santos de Assis L., Ratschen E. (2021). The influence of human–animal interactions on mental and physical health during the first COVID-19 lockdown phase in the UK: A qualitative exploration. Int. J. Environ. Res. Publ. Health.

[28] Randler C., Kalb N., Tryjanowski P. (2021). Sentiment Analysis of Comments of American Birders during Two Waves of the COVID-19 Pandemic Reveal More Negative Sentiments in the Context of Birding. Int. J. Environ. Res. Publ. Health.

[29] Kim E., Patterson S. (2022). The pandemic and gender inequality in academia. PS Political Sci. Polit..

[30] Carli L.L. (2020). Women, Gender equality and COVID-19. Gender in Management: Int. J..

[31] Craig L. (2020). Coronavirus, domestic labour and care: Gendered roles locked down. J. Sociol..

[32] Power K. (2020). The COVID-19 pandemic has increased the care burden of women and families. Sustain. Sci. Pract. Pol..

[33] UN Women (2020). The shadow pandemic: violence against women and girls and COVID-19. https://www.unwomen.org/en/news/in-focus/in-focus-gender-equality-in-covid-19-response/violence-against-women-during-covid-19.

[34] Mutz M. (2021). Forced adaptations of sporting behaviours during the Covid-19 pandemic and their effects on subjective well-being. Eur. Soc..

[35] Mutz M., Reimers A.K. (2021). Leisure time sports and exercise activities during the COVID-19 pandemic: a survey of working parents. Ger. J. Exerc. Sport Res..

[36] Michèle B., Choi S., Tripodi E., Broek-Altenburg E.v.d., Jamison J.C., Papageorge N.W. (2021). Unequal consequences of Covid 19: representative evidence from six countries. Rev. Econ. Househ..

[37] Wolfe K., Sirota M., Clarke A.D.F. (2021). Age differences in COVID-19 risk-taking, and the relationship with risk attitude and numerical ability. R. Soc. Open Sci..

[38] Pichlerová M., Önkal D., Bartlett A., Výbošťok J., Pichler V. (2021). Variability in forest visit numbers in different regions and population segments before and during the COVID-19 pandemic. Int. J. Environ. Res. Publ. Health.

[39] Randler C. (2021). An analysis of heterogeneity in German speaking birdwatchers reveals three distinct clusters and gender differences. Birds.

[40] Randler C., Diaz-Morales J.F., Jokimäki J., Ortiz-Pulido R., Staller N., De Salvo M., Tryjanowski P., Tsai J.S., de Almeida Barbosa R., Kaisanlahti-Jokimäki M.L., Kaisanlahti-Jokimäki M.L. (2023). Birding recreation specialization–A test of the factorial invariance in eight languages. J. Leisure Res..

[41] Hvenegaard G.T. (2002). Birder specialization differences in conservation involvement, demographics, and motivations. Hum. Dimens. Wildl..

[42] Sullivan B.L., Aycrigg J.L., Barry J.H., Bonney R.E., Bruns N., Cooper C.B., Damoulas T., Dhondt A.A., Dietterich T., Farnsworth A., Kelling S. (2014). The eBird enterprise: An integrated approach to development and application of citizen science. Biol. Conserv..

[43] Randler C. (2021). Users of a citizen science platform for bird data collection differ from other birdwatchers in knowledge and degree of specialization. Global Ecology and Conservation.

[44] Rice W.L., Mateer T.J., Reigner N., Newman P., Lawhon B., Taff B.D. (2020). Changes in recreational behaviors of outdoor enthusiasts during the COVID-19 pandemic: analysis across urban and rural communities. J. Urban Econ..

[45] Stenhouse A., Perry T., Grützner F., Rismiller P., Koh L.P., Lewis M. (2022). COVID restrictions impact wildlife monitoring in Australia. Biol. Conserv..

[46] Lee J.H., Scott D. (2004). Measuring birding specialization: A confirmatory factor analysis. Leisure Sci..

